# Prognostic value of non-invasive programmed ventricular stimulation after VT ablation to predict VT recurrences

**DOI:** 10.1007/s10840-024-01883-y

**Published:** 2024-08-16

**Authors:** Julian Müller, Ivaylo Chakarov, Karin Nentwich, Artur Berkovitz, Sebastian Barth, Felix Ausbüttel, Christian Wächter, Heiko Lehrmann, Thomas Deneke

**Affiliations:** 1 Department of Cardiology, Faculty of Medicine, University Heart Center Freiburg-Bad Krozingen,, University of Freiburg, Freiburg im Breisgau, Germany; 2 Clinic for Interventional Electrophysiology, Heart Centre Bad Neustadt, Von-Guttenberg-Straße 11, 97616 Bad Neustadt an Der Saale, Germany; 3 Department of Cardiology and Angiology, Philipps-University Marburg, Marburg, Germany; 4 Clinic for Electrophysiology, University Hospital of the Paracelsus Medical University, Klinikum Nuremberg, Campus South, Nuremberg, Germany

**Keywords:** VT ablation, Sudden cardiac death, Programmed stimulation

## Abstract

**Background:**

The prognostic value of (non)-invasive programmed ventricular stimulation (NIPS) to predict recurrences of ventricular tachycardia (VT) is under discussion. Optimal endpoints of VT ablation are not well defined, and optimal timepoint of NIPS is unknown. The goal of this study was to evaluate the ability of programmed ventricular stimulation at the end of the VT ablation procedure (PVS) and NIPS after VT ablation to identify patients at high risk for VT recurrence.

**Methods:**

Between January 2016 and February 2022, consecutive patients with VT and structural heart disease undergoing first VT ablation and consecutive NIPS were included. In total, 138 patients were included. All patients underwent NIPS through their implanted ICDs after a median of 3 (1–5) days after ablation (at least 2 drive cycle lengths (500 and 400 ms) and up to four right ventricular extrastimuli until refractoriness). Clinical VT was defined by comparison with 12-lead electrocardiograms and stored ICD electrograms from spontaneous VT episodes. Patients were followed for a median of 37 (13–61) months.

**Results:**

Of the 138 patients, 104 were non-inducible (75%), 27 were inducible for non-clinical VTs (20%), and 7 for clinical VT (5%). In 107 patients (78%), concordant results of PVS and NIPS were observed. After 37 ± 20 months, the recurrence rate for any ventricular arrhythmia was 40% (normal NIPS 29% vs. inducible VT during NIPS 66%; log-rank *p* = 0.001) and for clinical VT was 3% (normal NIPS 1% vs. inducible VT during NIPS 9%; log-rank *p* = 0.045). Positive predictive value (PPV) and negative predictive value (NPV) of NIPS were higher compared to PVS (PPV: 65% vs. 46% and NPV: 68% vs. 61%). NIPS revealed the highest NPV among patients with ICM and LVEF > 35%. Patients with inducible VT during NIPS had the highest VT recurrences and overall mortality. Patients with both negative PVS and NIPS had the lowest any VT recurrence rates with 32%. Early re-ablation of patients with recurrent VTs during index hospitalization was feasible but did not reveal better long-term VT-free survival.

**Conclusions:**

In patients after VT ablation and structural heart disease, NIPS is superior to post-ablation PVS to stratify the risk of VT recurrences. The PPV and NPV of NIPS at day 3 were superior compared to PVS at the end of the procedure to predict recurrent VT, especially in patients with ICM.

**Graphical Abstract:**

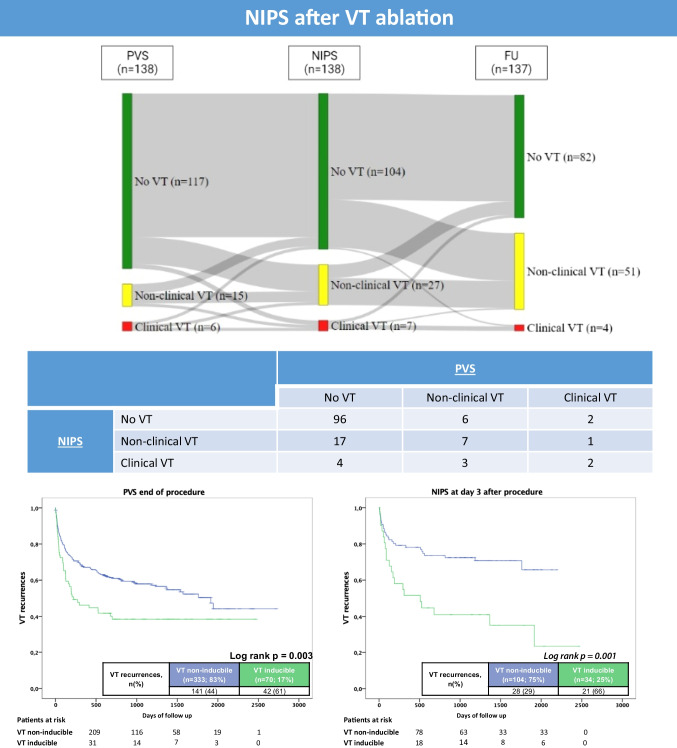

## Introduction

Ventricular tachycardia (VT) often complicates advanced stages of structural heart diseases (SHD) and is an important cause of sudden cardiac death (SCD) [1]. Catheter ablation of sustained VTs has shown to be an effective treatment option to reduce VT recurrences and prolong ICD shock-free survival [2–4]. Endpoints for ablation of VTs in the context of SHD are not well defined and typically include non-inducibility of the clinical VT or all VTs at programmed ventricular stimulation (PVS) during the index procedure. Still, modalities and intensivity of stimulation protocols at PVS are not clearly standardized, and VT recurrences may frequently occur during follow-up [5]. Possible reasons might be changes in antiarrhythmic drugs after the ablation procedure, the different autonomic tone during sedation/general anesthesia, the expansion of ablation lesions even after VT ablation due to disruption of microcirculation [6], and the probabilistic nature of the testing itself. Also, at the end of the ablation procedure sedation, acute ablation effects and hemodynamics of the patient may vary from short-term follow-up. To partially eliminate these cofounders, additional endpoints and methods are warranted to stratify patients according to the risk of VT recurrences. Non-invasive programmed ventricular stimulation (NIPS) via implantable defibrillator devices (ICD) several days after the ablation may identify patients with a high risk of VT recurrence in the subsequent year [7]. Differences of NIPS in ischemic and non-ischemic cardiomyopathies have not been evaluated clearly.

In the present study, we report our experience of the use of NIPS after VT ablation to assess the procedural outcome as well as to estimate the patients’ risk for long-term VT recurrences.

## Methods

### Study population

Consecutive patients with SHD who were referred for electrophysiological study and first ablation of VT between January 2016 and February 2022 were included in this study.

VT was mostly documented by ICD and if available by 12-lead electrocardiogram (ECG), ECG tele-monitoring, or in case of unstable course or during resuscitation by external defibrillator monitoring [8]. The definition of respective underlying cardiomyopathy was performed according to European guidelines [9, 10]. Patients with toxic cardiomyopathy, arrhythmia-induced cardiomyopathy, and primary valvular abnormalities were excluded. Patients with reduced LVEF and proven substrate but without comprehensible heart disease were rendered as “idiopathic.”

Standardized transthoracic echocardiographic examinations were usually performed before hospital discharge and admission, and values of left ventricular ejection fraction (LVEF) were retrieved before discharge to assess LVEF beyond the acute phase of VT. The documentation period lasted from index VT ablation until November 2022.

All patients gave written informed consent to all pre- and post-ablation diagnostics and the ablation procedure. The study was carried out according to the principles of the Declaration of Helsinki and was approved by the local medical ethics committee of the Heart Centre Bad Neustadt, Germany. All patients gave informed consent for participation in this retrospective analysis.

### Electrophysiological study

In most patients, VT ablation was performed under analgosedation (analgesia-first-based sedation) and in the fasting state using a continuous propofol infusion in conjunction with morphine derivatives. General anesthesia was only used when necessary, at the discretion of the operator, and only in minor part (< 5%). For all procedures, a high-density three-dimensional electroanatomic mapping system (CARTO 3, Biosense Webster, Diamond Bar, CA, USA; Ensite Precision, Abbott, St. Paul, MN, USA; Rhythmia, Boston Scientific, Natick, MA, USA) was used.

Our standard approach includes a high-density voltage map acquired with a high-density multipolar mapping catheter (Pentaray, Biosense Webster, Diamond Bar, CA, USA; Advisor HD Grid, Abbott, St. Paul, MN, USA; Intellamap Orion, Boston Scientific, Natick, MA, USA). Bipolar areas with voltage values ≤ 0.5 mV were defined as scar and low-voltage areas with values of ≤ 1.5 mV but > 0.5 mV as initially defined by Marchlinski et al. [11]. As additional criteria to identify abnormal ventricular tissue late potentials, local abnormal ventricular potentials and fractionated low amplitude potentials were annotated [12].

An epicardial approach was obtained in some cases using the percutaneous subxiphoid approach at the discretion of the operator. Prior to epicardial access, anticoagulation with non-vitamin-K oral antagonists (NOACs) was stopped the day before the procedure, and vitamin-K antagonists (VKAs) were stopped as appropriate in order to reach an INR level below 1.5, if the procedure could be planned.

All ablation procedures were performed according to our institutional standardized protocol by experienced operators. High-density mapping to characterize the underlying electrophysiological substrate in low-voltage areas and short episodes of inducible VT for activation and entrainment mapping was performed. Ablation targets were as follows: (1) identified critical VT isthmuses and (2) late potentials in low-voltage areas. All ablations were performed with irrigated RF ablation of 45W and standard lesion parameters. The endpoint of ablation procedures was evaluated at least once at the end of the procedure: (1) partial short-term success if non-inducibility of the clinical VT but other VTs were still inducible; (2) complete short-term success as elimination of any inducible VT; (3) ablation of all tagged abnormal electrograms. At the end of the procedure, usually, PVS was performed with stimuli 2 ms in duration at twice diastolic threshold up to 4 extrastimuli at 2 basic drive cycle lengths (500 and 400 ms), at the right ventricular apex, and at at least one left ventricular site. Coupling intervals of extrastimuli were decreased in 20-ms intervals until refractoriness or induced VT.

### (Non)-invasive programmed stimulation

The reason for deferring NIPS was repeated non-inducibility at the beginning and during the procedure, spontaneous VT recurrence between VT ablation and before planned NIPS, and upon patients’/operators’ preference. NIPS was performed in all clinically stable patients without VT recurrences during the initial post-ablation phase via implanted ICD several days after ablation before hospital discharge. In most patients, antiarrhythmic drug therapy was discontinued except beta-blockers. In the fasting state under beta-blocker therapy in the awake state from the right ventricular ICD lead, drive trains of 500 and 400 ms with up to 4 extrastimuli were applied as feasible based on ICD specifications. Coupling intervals of extrastimuli were decreased in 20 ms intervals until refractoriness or induced VT. The output was set at 5 V/2 ms. In patients with biventricular pacing devices, also, trains of 500 and 400 ms with up to 4 extrastimuli were applied via LV-only pacing as feasible based on ICD specifications. Only patients with ICD feasible of NIPS were included. Sedation with midazolam was used only in case of induction of any VT for the purpose of ICD shock.

The endpoint “clinical VT inducible” was categorized, if any sustained monomorphic VT was induced matching the spontaneous VTs. “Non-clinical VT inducible” was categorized if only sustained monomorphic or polymorphic VTs were induced not matching the clinical VTs in QRS morphology and cycle length. “No VT inducible” was categorized if no sustained monomorphic VT was inducible. In the case of VT induction during NIPS, ICD programming was adopted accordingly to record all induced VT-cycle lengths.

### Clinical follow-up and endpoint

Patient data including all adverse events and deaths were collected until discharge and documented. ICD programming after VT ablation typically included the slowest clinical and/or induced VT zone. VT with a CL equal to or longer than the clinical VT documented via ICD or having the same QRS morphology on event 12-lead ECG was considered recurrent clinical VT recurrence.

The endpoint was the recurrence of any sustained ventricular arrhythmia (VA) documented either by ICD interrogations during follow-up in most cases or ECG.

### Statistical methods

Quantitative data are presented as mean ± standard error of mean (SEM), median and interquartile range (IQR), and ranges depending on the distribution of the data and were compared using Student’s *t* test for normally distributed data or the Mann–Whitney *U* test for nonparametric data. Deviations from a Gaussian distribution were tested by the Kolmogorov–Smirnov test. Spearman’s rank correlation for nonparametric data was used to test univariate correlations. Qualitative data are presented as absolute and relative frequencies and compared using the Chi^2^ test or Fisher’s exact test, as appropriate.

Continuous variables were evaluated by logistic regression; categorical variables were analyzed by contingency tables. Univariate regression analysis was performed for significant and clinically relevant variables. The result of a statistical test was considered significant for *p* < 0.05, and *p* values ≤ 0.1 were defined as a statistical trend. SAS, release 9.4 (SAS Institute Inc., Cary, NC, USA) and SPSS (Version 25, IBM Armonk, New York, USA) were used for statistics. The Sankey diagram was designed using R Studio with the packages “Rcmdr,” “dplyr,” and “NetworkD3” as well as the BioRender software.

## Results

### Study population

**Fig. 1:**
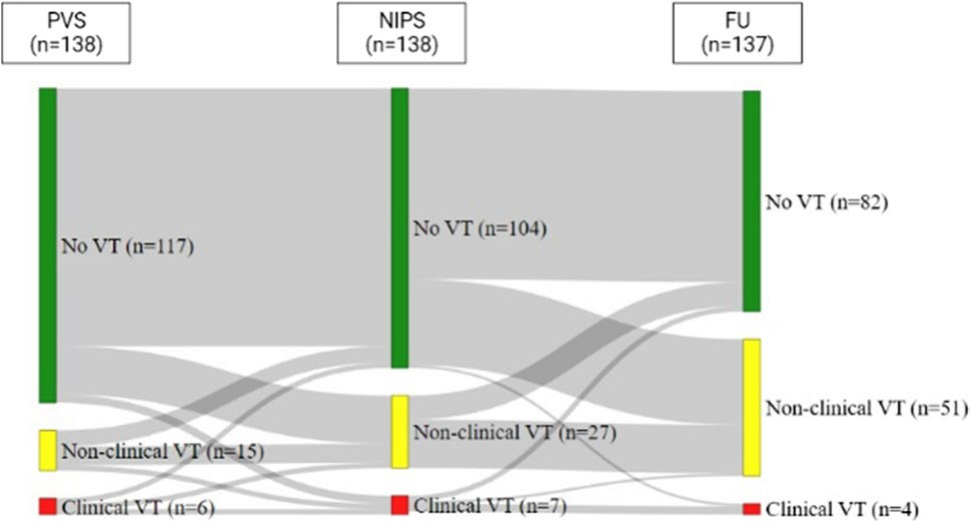
Sankey diagram illustrating concordant and different results of PVS, NIPS, and spontaneous VT recurrences during follow-up. Green = no VT inducible; yellow = non-clinical VT inducible; red = clinical VT inducible

A total of 138 patients out of a total group of 446 structural VT patients underwent NIPS with a median of 3 (1–5) days after VT ablation at our institution and were included (Fig. 1). Reasons for not performing NIPS were patients/physicians’ preference or repeated non-inducibility, defined as non-inducibility at the beginning and the end of the ablation procedure in most cases.

The most common underlying structural diseases were ICM (56%), followed by idiopathic dilated CM (18%), and myocarditis (11%), whereas 6 patients (4%) showed no SHD and were therefore rendered as idiopathic. Mean LVEF was 37 ± 14. Implanted devices were VVI-ICD in 68 patients (49%), dual chamber ICD in 30 patients (22%), and CRT-Ds in 40 patients (29%) mostly for secondary prevention. Baseline characteristics were comparable between all groups undergoing NIPS. Detailed baseline characteristics can be seen in Table 1.

**Table 1:** Baseline characteristics

Characteristic	NIPS prior discharge (*n* = 138; 100%)	Normal NIPS (*n* = 104; 75%)	NIPS non-clinical VT inducible (*n* = 27; 20%)	NIPS clinical VT inducible (*n* = 7; 5%)	*p* value
Age, median	63 ± 13	64 ± 13	60 ± 14	67 ± 10	0.339
Males, *n* (%)	119 (86)	90 (87)	22 (82)	7 (100)	0.441
Cardiovascular risk factors, *n* (%)					
Arterial hypertension	111 (80)	86 (83)	20 (74)	5 (71)	0.499
Diabetes mellitus	38 (28)	31 (30)	35 (19)	2 (29)	0.503
Hyperlipidemia	102 (74)	78 (75)	20 (74)	4 (57)	0.581
Smoking	44 (32)	35 (34)	8 (31)	1 (14)	0.561
Cardiac family history	25 (18)	18 (17)	6 (22)	1 (14)	0.810
Comorbidities, *n* (%)					
Atrial fibrillation	52 (38)	39 (38)	10 (37)	3 (43)	0.958
Stroke	16 (12)	14 (14)	2 (7)	0 (0)	0.420
Chronic kidney disease	72 (52)	58 (59)	10 (37)	4 (57)	0.214
COPD	15 (11)	11 (11)	4 (15)	0 (0)	0.523
Structural heart disease, *n* (%)					
Ischemic cardiomyopathy	77 (56)	60 (58)	12 (44)	5 (71)	0.324
Dilated cardiomyopathy	25 (18)	17 (16)	7 (26)	1 (14)	0.497
Myocarditis	15 (11)	12 (12)	2 (7)	1 (14)	0.792
Sarcoidosis	4 (3)	3 (3)	1 (4)	0 (0)	0.873
ARVC	10 (7)	7 (7)	3 (11)	0 (0)	0.552
HCM	1 (1)	1 (1)	0 (0)	0 (0)	0.848
Idiopathic	6 (4)	4 (4)	2 (7)	0 (0)	0.610
NCCM	1 (1)	1 (1)	0 (0)	0 (0)	0.848
Medication at admission, *n* (%)					
Beta-blocker	120 (87)	93 (92)	22 (88)	5 (71)	0.188
Amiodarone	43 (31)	28 (27)	12 (48)	3 (43)	0.126
Other AAD	4 (3)	1 (1)	3 (12)	0 (0)	**0.015**
LVEF (%)	37 ± 14	37 ± 13	38 ± 16	38 ± 15	0.847
Electrical storm, *n* (%)	45 (33)	30 (29)	11 (41)	4 (57)	0.183
Type of ICD, *n* (%)					
ICD	98 (71)	80 (77)	16 (59)	2 (29)	**0.008**
CRT-D	40 (29)	24 (23)	11 (41)	5 (71)	
ICD indication, *n* (%)					
Primary prevention	46 (33)	34 (33)	10 (37)	2 (29)	0.879
Secondary prevention	92 (67)	70 (67)	17 (63)	5 (71)	

*AAD*, antiarrhythmic drug; *ARVC*, arrhythmic right ventricular disease; *COPD*, chronic obstructive pulmonary disease; *CRT-D*, cardiac resynchronization therapy-defibrillator; *HCM*, hypertrophic cardiomyopathy; *HOCM*, hypertrophic obstructive cardiomyopathy; *ICD*, implantable cardioverter defibrillator; *LVEF*, left ventricular ejection fraction

### Procedural characteristics and acute procedural outcomes

Procedural data from the VT ablation procedures are summarized in Table 2. During the procedure, a median of 1 (0–2) VTs were inducible with higher numbers in patients with recurrent clinical and non-clinical VTs. All patients underwent endocardial mapping with epicardial ablation in 25%. Ablation failure and use of catecholamines were higher in patients with inducible VTs during NIPS with consecutive higher rates of amiodarone medication at discharge (Table 2). At the end of the procedure, 117 patients (85%) had no VT inducible, whereas 15 patients (11%) had non-clinical VT, and 6 patients (4%) had clinical VT inducible during PVS (see Fig. 1). Of note, those patients without pre-discharge NIPS had comparable rates of inducible clinical (4% vs. 5%) and any VT (15% vs. 20%) at the end of the procedure.

**Table 2:** Procedural data and intraprocedural success

Characteristic	Normal NIPS (*n* = 104; 75%)	Non-clinical VT during NIPS (*n* = 27; 20%)	Clinical VT during NIPS (*n* = 7; 5%)	*p* value
Epicardial ablation, *n* (%)	24 (23)	7 (26)	3 (43)	0.494
Non-inducible with PVS at beginning, *n* (%)	21 (20)	0 (0)	0 (0)	**0.001**
VTs inducible, *n*/patient	2 (1–3)	3 (1–5)	3 (1–5)	**0.056**
Clinical VT CL (ms)	347 ± 88	349 ± 89	349 ± 37	0.988
Procedural duration (min)	145 ± 41	170 ± 59	236 ± 74	**0.001**
Fluoroscopy duration (min)	14.1 ± 9.6	28.4 ± 11.5	29.7 ± 8.0	**0.001**
Ablation time (min)	28.8 ± 15.8	35.2 ± 21.2	43.0 ± 16.0	**0.044**
Ablation failure, *n* (%)	2 (2)	2 (7)	2 (29)	**0.001**
Partial ablation success, *n* (%)	102 (98)	25 (93)	5 (71)	**0.001**
Full ablation success, *n* (%)	96 (92)	18 (69)	3 (43)	**0.001**
Hemodynamic not tolerated VT, *n* (%)	24 (23)	11 (41)	1 (14)	0.135
Catecholamine, *n* (%)	8 (8)	2 (8)	2 (29)	0.163
General anesthesia, *n* (%)	1 (1)	0 (0)	0 (0)	0.848
Beta-blocker at discharge, *n* (%)	103 (99)	26 (96)	7 (100)	0.539
Amiodarone at discharge, *n* (%)	16 (15)	13 (48)	3 (43)	**0.001**

*CL*, cycle length; *CT*, computer tomography; *ES*, electrical storm; *MRI*, magnet resonance imaging; *PES*, programmed electrical stimulation; *VT*, ventricular tachycardia

### (Non)-invasive programmed ventricular stimulation

NIPS was performed in all 138 patients via the ICD. No VT was inducible in 104 patients (75%), a non-clinical VT in 27 (20%), and clinical VT in 7 patients (5%) (Fig. 1). Induction of VT was achieved with 1 extrastimulus in 4 patients (3%), with 2 extrastimuli in 10 patients (7%), and with 3 extrastimuli in 20 patients (14%). Of the 34 induced VTs, 16 could be terminated with ATP, 16 were terminated with DC shock, and 2 were self-terminating. Interestingly, patients undergoing NIPS had a shorter time to discharge from VT ablation (median of 5 days) compared to those without (median of 6 days).

Of those 6 patients with clinical VT still inducible at the end of the procedure, 4/6 had continued pre-existing amiodarone treatment, and of the 15 patients with still inducible non-clinical VT, amiodarone was continued in 5 (with already pre-existing amiodarone treatment) and held in 2 others until NIPS. The rest did not have AAD, and no new therapy was initiated between ablation and NIPS. Of the 34 patients with VTs inducible during NIPS, 16 (47%) were discharged with amiodarone. No other AAD was used during discharge. NIPS-guided AAD adjustment was performed in 19 patients with discontinuation in 13 patients with normal NIPS, in 2 patients with positive NIPS initiated, and in 4 patients with positive NIPS adjusted. Furthermore, ICD programming was adjusted in 15 patients in which VTs with longer CL than previously known VTs were induced.

### Follow-up VT recurrence and mortality

**Fig. 2:**
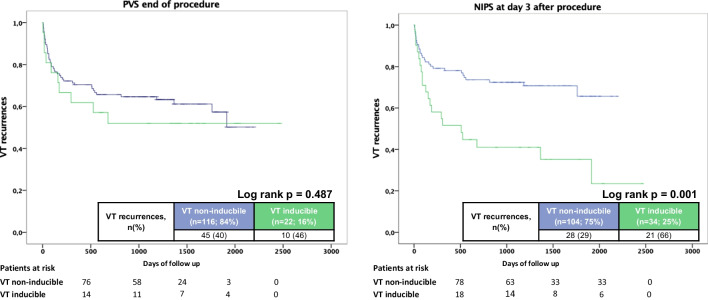
Kaplan–Meier analysis of any ventricular tachycardia (VT) recurrences according to programmed ventricular stimulation (PVS) result after ablation procedure (left panel) and non-invasive programmed stimulation (NIPS) at day 3 (right panel)

Median follow-up time was 37 (13–61) months. The recurrence rate for any ventricular arrhythmia (VA) during follow-up was 40%. Overall recurrence rates were significantly lower among patients with normal NIPS (normal NIPS 29% vs. inducible VT during NIPS 66%; log-rank *p* = 0.001) (Fig. 2). Recurrence rates of clinical VTs were low with 3% with higher recurrences among patients with inducible VT during NIPS (normal NIPS 1% vs. inducible VT during NIPS 9%; log-rank *p* = 0.045). Of note, patients without NIPS before discharge revealed higher VT recurrences (53% vs. 40%; log-rank *p* = 0.008).

Overall mortality during follow-up was 10%. Patients with positive NIPS had a higher mortality rates than patients with normal NIPS (18% vs. 7%; log-rank *p* = 0.001). Patients with clinical VT inducible during NIPS had the highest mortality, followed by non-clinical VTs, whereas patients with normal NIPS had the lowest mortality (29% vs. 16% vs. 7%; log-rank *p* = 0.001). There was no significant difference of VT recurrences between patients induced with 1 (50%), 2 (60%), or 3 (65%) extrastimuli (*p* = 0.845).

### Early vs. late ventricular stimulation

Concordant results of PVS at the end of the ablation and NIPS were observed in 105 of 138 patients (76%) (Table 3 and Fig. 2). The positive (PPV) and negative predictive values (NPV) to predict late VT recurrences of early PVS were 46% (10/22) and 61% (71/116), respectively. The PPV and NPV of NIPS at day 3 were 65% (22/34) and 68% (71/104), respectively. Figure 2 shows the long-term VT recurrence rates according to early PVS and NIPS at day 3.

**Table 3:** Comparison between results of PVS and NIPS

		PVS		
		No VT	Non-clinical VT	Clinical VT
NIPS	No VT	***96***	6	2
	Non-clinical VT	17	***7***	1
	Clinical VT	4	3	***2***

Eighteen percent of all patients undergoing NIPS (21/117) were rendered non-inducible at PVS at the end of the procedure, but VT was again inducible at NIPS. These patients had significantly worse VT recurrence rates of 71% (15/21) compared to patients who were rendered non-inducible at PVS at the end of the procedure and NIPS at day 3 (31%; 30/96) (log-rank *p* = 0.002), irrespective of higher amiodarone therapies (35% vs. 15%; *p* = 0.031).

### Predictive value according to patient profiles

**Fig. 3:**
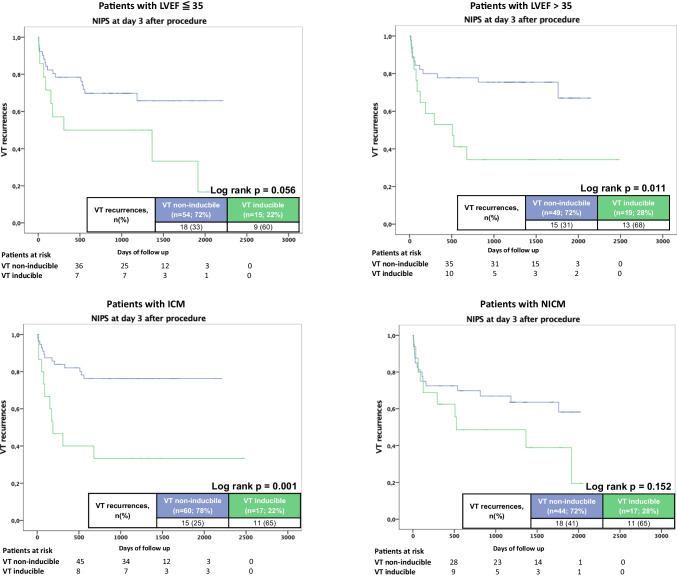
Kaplan–Meier analysis of any ventricular tachycardia (VT) recurrences according to non-invasive programmed stimulation (NIPS) at day 3 depending on LV function (upper panels) and structural heart disease (lower panels)

The predictive values of PVS and NIPS at day 3 after the procedure for VT recurrences were further analyzed in different subgroup on the basis of LVEF and underlying cardiomyopathy. NIPS revealed higher NPV compared to PVS irrespective of LVEF (36/42; NPV 67% vs. 37/62; NPV 60% for LVEF < 35 and 34/49; NPV 69% vs. 34/54; NPV 63% for LVEF > 35). Figure 3 illustrates the Kaplan–Meier curves for VT recurrences stratified for LV function and VT inducibility at PVS and NIPS.

Among patients with ICM, NIPS had higher NPV (45/60; NPV 75%) than PVS (44/66; NPV 75%) as well as among patients with NICM (26/44; NPV 59% vs. 27/50; NPV 54%). Figure 3 illustrates the Kaplan–Meier curves for VT recurrences stratified for underlying heart disease and VT inducibility at the end of the procedure and NIPS at day 3.

### Repeat ablations during the initial hospital stay and its influence on long-term outcomes

Of the 34 patients with positive NIPS, 7 (21%) underwent repeat procedures prior to hospital discharge at a mean of 2.3 ± 1.7 days after the index procedure (4/7 with non-clinical VT; 3/7 with clinical VT). Four out of seven had septal substrate and underwent redo focusing on septal VT, 2 underwent epicardial approach after initial endocardial only, and 1 patient underwent redo endocardial only. The reason to undergo recurrent ablation during the same stay was an electrical storm in 5 patients and planned bipolar septal ablation in the other two. Five out of seven patients underwent a redo NIPS, of which 4 were non-inducible and a non-clinical VT in 1 patient. Patients undergoing NIPS-directed early repeat ablations had comparable VT recurrence rates than NIPS-positive patients without repeated ablations (overall group 71% redo vs. 63% no redo; *p* = 0.338) during the initial hospitalization.

## Discussion

This study reports a single-center experience comparing PVS and NIPS after VT ablation and its prognostic implications on long-term VT recurrences. The major findings of this study are as follows: (1) a significant portion of all patients reveal new non-clinical VTs as recurrence during follow-up; (2) concordant results of PVS at the end of procedure and NIPS at day 3 after ablation were observed in 3/4 of all patients; (3) NIPS has a superior NPV compared to PVS irrespective of type of underlying SHD; (4) NPV of NIPS is best in patients with ICM and LVEF > 35%; (5) early redo procedures after intrahospital VT recurrence are safe and feasible but are not associated with better long-term outcome.

### Non-inducibility as clinical endpoint

In the last decade, several randomized controlled trials emerged reporting the superiority of catheter ablation compared to antiarrhythmic drugs in preventing VT recurrences and ICD therapies among patients with SHD [4, 13, 14]. However, long-term results after ablation remain improvable with reported recurrence rates of up to 50% during follow-up [14–16]. Therefore, optimal endpoints of catheter ablation effectively predicting VT recurrence during follow-up are still warranted. Today, in most centers, PVS at the end of the procedure is performed to assess acute ablation success. Accordingly, current guidelines recommend VT non-inducibility as the endpoint [17, 18] of VT ablation procedures. On the other hand, patients with still inducible VTs are considered high-risk patients for VT recurrences [19], and repeat ablation may be considered in these patients. However, PVS at the end of the procedure has several limitations reducing its prognostic value. Some patients with poor hemodynamics at the end of a long ablation procedure may not tolerate VT inducibility testing at the end, and it is therefore avoided or abbreviated at the discretion of the operator. But even when PVS is performed rigorously at the end of each procedure, its sensitivity may be hampered by altered autonomic tone due to deep sedation or general anesthesia, subsequent changes in antiarrhythmic drug medications, regression and maturation of ablation lesions, imperfect reproducibility [7], and most importantly, the probabilistic nature of VT induction, meaning the more you stimulate, the more likely VTs are to be induced. Accordingly, studies evaluating the prognostic value of PVS at the end of the ablation procedure delivered conflicting evidence [20–22]. Acute non-inducibility, despite being a necessary endpoint for the ablation procedure, may not adequately identify patients at the lowest risk for recurrences, and inducibility may be altered within the days afterwards [23]. In our series, for example, 4 patients with inducible clinical VT during NIPS had no VT inducible at the end of the procedure using aggressive PVS.

### Prognostic value of additional NIPS after VT ablation

Additional risk stratification tools are needed, and several additional endpoints apart from acute non-inducibility have been proposed. Remote NIPS performed several days after the ablation procedure but, prior to hospital discharge, has been evaluated as an additional marker for VT recurrence after ablation. This procedure has been validated in consecutive patients with SHD after VT ablation who were tested with NIPS after a median of 3 days post VT ablation. The authors found that those patients with clinical VT still inducible at NIPS had a significantly decreased 1-year VT-free survival (< 30%) compared to those with no VT inducible (85%) [7]. Slightly higher recurrence rates were observed within our cohort; however, our population contained a higher part of NICM patients and longer follow-up period.

In our study, 29 patients showed contrary results comparing NIPS and PVS mostly driven by VT inducibility of VTs at NIPS. It is important to know that some patients still inducible at PVS were non-inducible at NIPS, and none of these patients had recurrences during long-term follow-up.

Our study reports that the prognostic power of PVS and especially NIPS early after ablation is best in patients with LVEF > 35 and in patients with ICM. In line with these results, among patients with NICM, severely reduced LVEF as well as persistent VT inducibility at NIPS were confirmed as the only independent predictors of VT recurrence during long-term follow-up [24]. Among patients with severely reduced LVEF, the prognostic relevance of NIPS is thought to be decreased due to high recurrence rates irrespective of the acute ablation outcome [21]. Although the patient collective in our study was well balanced between ICM and NICM, the NPV of NIPS stratified according to LVEF was satisfyingly independent to LVEF < 35% and regardless of the underlying cardiomyopathy.

The optimal timing of NIPS after VT ablation is still not clear, and recently, Oloriz et al. reported that NIPS performed after a median of 6 days after VT ablation had higher PPVs and NPVs than PVS at the end of the procedure [25]. Based on their observation, the authors hypothesized that repeated ablation should be performed, if the clinical VT is still inducible. NIPS-guided repeat ablation in case of inducibility of clinical VT was associated with a significantly lower risk for subsequent recurrences [26]. Our study could not show this relation even though at least numerically lower VT recurrence rates could be observed in patients with early repeat procedure in case of recurrent VT. Furthermore, recurrences of clinical VTs during NIPS were significantly lower in our study (5% vs. 21% in Muser et al.) [26].

### Clinical implications

Although we could not show a significant benefit of overall early repeat ablation for patients with positive NIPS studies, it might be reasonable in case of suspicion of incomplete ablation. Especially in patients with recurrent VTs and suspicion of epicardial origin after endocardial-only previous ablation early re-ablation might be feasible. The positive effect of early repeat ablation might be missed in this study due to the small sample size and the “negative selection” of patients with the highest risk of VT recurrence. Until clear identification of potential “responders” for repeated NIPS-guided re-ablation, the potential costs and risks of a prolonged hospital stay and repeat sedation in a “one size fits all” setting should be critically discussed.

Induction of non-clinical, previously unknown VT morphologies is reported to be associated with higher recurrence rates, which is concordant with the results of our study. This study also showed that those patients with clinical VT or at least any VT inducible during NIPS had the highest mortality rates. If this is primarily related to arrhythmic deaths, it is beyond the scope of the study. However, taking together the longer procedure and ablation times and the higher rates of epicardial ablations among those patients with clinical VTs, it seems reasonable to think that ablation was not successful due to a more complex underlying substrate. In ICM patients, a cycle length of > 300 ms of persistent VTs was associated with VT recurrences, whereas induction of fast VTs with a cycle length of < 230 ms might be nonspecific [27]. Escalation of AAD therapy seems to be inferior to repeat ablation as shown by the VANISH trial [14].

Patients with negative PVS and NIPS study showed VT recurrences in 32% during the follow-up period. This is higher than previously reported data; however, the follow-up period was significantly longer in our study [7, 25], and more NICM patients were included. Based on these results, it can be speculated that subsets of patients with negative NIPS, especially with ICM, might benefit from ICD programming similar to primary prevention purposes, as previously discussed by Oloriz et al. and Moss et al. [25, 28]. Also, as NPV in ICM with EF > 35% is best, this cohort may benefit from early repeat programmed stimulation if considering not to implant an ICD during follow-up.

## Study limitations

This study is a single-center, non-randomized study representing the experience of a high-volume EP center specifically dedicated to VT patients. We serve as a tertiary referral center for VT ablations, and as such, it is possible that there is a referral bias that may limit the generalizability of our results. The major limitation of this study is that not all consecutive patients underwent NIPS because of several reasons and differences between those who did and who did not exist as shown in Table 1. Although patients with NIPS had comparable acute procedural success rates compared to those without, higher VT recurrence rates and longer times from VT ablation to discharge might indicate a sicker patient population of those patients without NIPS. The distinction between clinical and non-clinical VT might be difficult as some patients do not have 12-lead ECGs of clinical VTs during follow-up. However, 12-lead ECGs were available in most of the cases, and if not, recorded ICD electrograms were used to identify clinical VT [29]. Prolongation between ablation and NIPS could influence predictive values; however, discharge from hospital during clinical practise might be even earlier than 3 days after VT ablation. The study represents a patient cohort with advanced heart failure and severely reduced left ventricular function. Patients with an earlier disease state may be different from those included in this study. The outcomes of PVS and NIPS only represent the current disease stage. Disease progression goes hand in hand with changes in the electrical milieu, and PVS/NIPS might not be able to evaluate the risk for VT in patients with progressive cardiomyopathy resulting in higher VT recurrences and lower overall predictive values due to longer follow-up times in our study compared to previous ones [7, 25, 26]. This was a retrospective analysis with its typical limitations.

## Conclusions

NIPS several days after VT ablation is a feasible tool to estimate the risk for VT recurrences with better PPV and NPV than intraprocedural PVS. Twenty-two percent of all patients showed inconsistent findings between PVS after ablation and NIPS with re-inducibility of VT despite complete non-inducibility at the timepoint of VT ablation and even higher VT recurrence rates. Early repeat procedures guided by positive NIPS studies at day 3 after the index procedure in our study did not result in better VT freedom.

## Data Availability

Data is available from the corresponding author upon reasonable request.
